# Wild-type TTR amyloidosis among patients with unexplained heart failure and systolic LV dysfunction

**DOI:** 10.1371/journal.pone.0254104

**Published:** 2021-07-09

**Authors:** Sorel Goland, Igor Volodarsky, Yacov Fabricant, Shay Livschitz, Sagi Tshori, Valeri Cuciuc, Liaz Zilberman, Irena Fugenfirov, Valeri Meledin, Sara Shimoni, Sagie Josfberg, Jacob George

**Affiliations:** 1 The Heart Institute, Kaplan Medical Center, Rehovot, Israel; 2 Hebrew University and Hadassah Medical School, Jerusalem, Israel; 3 Department of Genetics, Kaplan Medical Center, Rehovot, Israel; Univeristy of Tennessee, UNITED STATES

## Abstract

**Aim:**

Transthyretin cardiac amyloidosis (ATTR-CA) is an increasingly recognized cause of heart failure (HF) with preserved left ventricular ejection fraction (LVEF), typically presenting as restrictive cardiomyopathy. The potential co-existence of ATTR-CA with systolic heart failure has not been studied. The aim of this study is to describe the prevalence of ATTR-CA and its clinical characteristics in HF patients with reduced LVEF.

**Methods:**

Patients with an unexplained cause of LV systolic dysfunction were screened for ATTR-CA by a ^99m^Tc-PYP planar scintigraphy. Patients in whom presence of ≥ 2 uptake was confirmed by SPECT imaging were included. Their clinical, laboratory and echocardiographic data were collected.

**Results:**

Out of 75 patients (mean age 65±12 years, LVEF 35.8±7.9%) included in this study, 7 (9.3%) patients (mean age 75±6 years, LVEF 32.0±8.3%) had ATTR-CA. Patients with ATTR-CA were more symptomatic at diagnosis (NYHA FC 3–4 (86% vs 35% (p = 0.03)) and had a more severe clinical course evident by recurrent hospitalizations for HF, and a need for intravenous diuretic treatment (p = 0.04 and p<0.01, respectively) at follow-up, compared with patients with no ATTR-CA. Patients with ATTR-CA had similar LVEF but a clear trend for larger LV mass index (157.1±60.6 g/m^2^ vs. 121.0±39.5 g/m^2^, p = 0.07) and a larger proportions of ATTR-CA patients had IVS thickness >13 mm (57.1% vs 13.1%, p = 0.02) as compared to HF patients with no ATTR-CA.

**Conclusion:**

In our study, a meaningful percentage of patients with unexplained LV dysfunction had a co-existing ATTR-CA indicating that the clinical heterogeneity of ATTR-CA is much broader than previously thought.

## Introduction

Transthyretin amyloidosis cardiomyopathy (ATTR-CA) is a progressive infiltrative disease caused by extracellular deposition of misfolded transthyretin protein (TTR) in the myocardium presenting with progressive heart failure [1,2]. Based on the type of transthyretin protein, ATTR-CA is divided into a hereditary form (hATTR) caused by the presence of TTR mutations and a non-hereditary form or wild type (wtATTR) caused by age-related instability of wild-type TTR [2]. hATTR cardiomyopathy is often associated with extracardiac manifestations such as peripheral neuropathy and/or autonomic neuropathy, while patients with wtATTR present predominantly with isolated cardiac involvement, and wtATTR is considered an increasingly recognized cause of heart failure in older individuals [3–5]. ATTR–CA is a relatively rapidly progressive cardiomyopathy associated with poor prognosis. Therefore, early diagnosis and interventions using the latest available treatments are important [6–11]. Recently, a non-biopsy diagnosis of cardiac transthyretin amyloidosis became validated. Radionuclide bone scintigraphy with high sensitivity and specificity (99% and 86%, respectively) for cardiac ATTR amyloid [12] is a reliable tool for the diagnosis of ATTR cardiomyopathy. Phenotypic heterogeneity of the disease often leads to delayed or misdiagnosis with a number of cardiovascular conditions, mostly left ventricular hypertrophy caused by hypertensive cardiomyopathy and aortic stenosis (AS) [13,14]. ATTR-CA is traditionally considered a type of restrictive cardiomyopathy with preserved LVEF. However, cardiac involvement with a progressive reduction in LV function has been sporadically described among patients recently diagnosed with ATTR-CA as well as among patients with systolic heart failure and LV hypertrophy [1,15,16]. We hypothesized that ATTR can be found among a subset of patients with systolic heart failure irrespective of classical features considered as pathognomonic for ATTR-CA. Therefore, the aim of our study was to define the prevalence and clinical phenotype of ATTR-CA among patients with heart failure and unexplained impaired LV function.

## Methods

The protocol was approved by the Kaplan Medical Center ethics committee and all participants gave an informed written consent.

### Patients

A total of 404 patients with heart failure (HF) and LV systolic dysfunction (LVEF<50%) who presented in the Outpatient Heart Failure Clinic at Kaplan Medical Center were prospectively recruited between April 2017 to June 2019. Patients were systematically followed up until June 2020, the date of censoring. Heart failure with systolic dysfunction was defined as HF with either reduced (HFrEF) or mid-range (HFmrEF) left ventricular ejection fraction (LVEF) according to the ESC guidelines on diagnosis and management of heart failure 2016 (HFrEF is defined as LVEF <40%, and HFmrEF as LVEF 40–49%) [17]. Patients enrolled in this prospective study underwent an extensive workup including detailed history and physical examination, electrocardiography (ECG), echocardiography, and coronary artery angiography to rule out coronary artery disease. Patients with reduced LV function and HF of any known etiology (coronary artery disease, myocarditis, significant valvular disease, chemotherapy, or tachycardia related), prior diagnosis of plasma cell dyscrasia or AL amyloidosis were excluded.

All patients with LV systolic dysfunction (LVEF<50%) and unexplained cause of HF underwent a ^99m^Tc -Pyrophosphate Scintigraphy (^99m^Tc-PYP) scintigraphy.

### ^99m^Tc -Pyrophosphate scintigraphy

Anterior and lateral 750 K count views of the chest were acquired approximately 1 hour after 10 mCi ^99m^Tc-PYP injection using either ECAM or Symbia T2 cameras (Siemens) with a 256x256 matrix in accordance with the "ASNC practice points" (https://www.asnc.org/Files/Practice%20Resources/Practice%20Points/ASNC%20Practice%20Point-99mTechnetiumPyrophosphateImaging2016.pdf).

Blood pool activity was excluded, with use of SPECT imaging in suspected positive patients as was recently established in the ASNC/AHA/ASE/EANM/HFSA/ISA/SCMR/SNMMI Expert Consensus Recommendations [18,19]. Patients were imaged 1–2 hours after injection of 20 mCi 99mTc-99mTc-PYP using a CZT multidetector camera (D-SPECT Cardio, Spectrum Dynamics) with energy window set to 140±14 keV. Patients were pre-scanned for 60 sec for positioning of the heart, followed by a 5 min planar acquisition using 256x256 matrix. Patients were pre-scanned again, followed by gated SPECT acquisitions (10 min, 8 gating bins, 128x128 matrix). Two nuclear cardiologists/radiologists blinded to the patients’ clinical status independently evaluated cardiac retention of ^99m^Tc-PYP using a semiquantitative visual scoring method (0 = no uptake, 1 = uptake less than ribs, 2 = uptake equal to ribs, 3 = uptake greater than ribs) [12,14]. Patients with an inconclusive 99mTc-PYP scintigraphy patients and those who had a positive planar scan, but were not able to undergo an additional SPECT scan to exclude blood pool for different reasons were also excluded from the analysis. Patients with presence of ≥ 2 uptake by planar scan that was confirmed by SPECT 99mTc-PYP scintigraphy were defined as ATTR-CA positive.

### Echocardiography and electrocardiography

Left ventricular (LV) dimensions, LVEF and LV mass were measured according to the American Society of Echocardiography recommendations and were assessed by conventional Doppler imaging and TDI [20,21]. The following parameters were measured by pulse-wave Doppler: peak velocities of early (E) and late (A) diastolic filling, and deceleration time. TDI measured early (E′) and late diastolic velocity (A′) and systolic velocity (S′). Raw data were stored digitally as DICOM cine loops and transferred for offline analysis to a workstation with the EchoPAC software (PC Dimension version 5.0.1; GE Vingmed Ultrasound, Horten, Norway). For 2D speckle tracking, the LV myocardium was imaged with a frame rate >50 Hz. Measurements of 2D strain (2DS) and strain rate (SR) were performed by offline semiautomatic analysis. The endocardial border was semi manually traced, and the myocardial region of interest was automatically identified by the software package. 2DS and SR were measured in the three apical views (which were then averaged) to determine longitudinal 2DS (LGS) and SR. The relative apical sparing index was defined using the equation: average apical LS/(average basal LS + mid-LS) [22]. Voltage-to-mass ratio proposed by Rapezzi et al [1] was calculated as Sokolow index divided by LV mass index as a more sensitive index than ECG characterizing amyloid cardiomyopathy was measured only in patients without RBBB and paced rhythm on ECG.

### Non-biopsy diagnosis of patients with ATTR-CA

Patients with a positive ^99m^ Tc-PYP scintigraphy underwent additional workup to exclude primary amyloidosis (AL) (serum free light chain essay and serum/urine immunofixation) and were assessed for extracardiac manifestations. The patient clinical data including comorbidities (hypertension, smoking status, diabetes, obesity, atrial fibrillation, cerebrovascular disease, and cardiac pacemaker implantation history), medications, laboratory, and electrocardiographic recordings were also collected. Information regarding NYHA functional class (NYHA FC), hospitalizations, outpatient unit visits for intravenous diuretics and daycare heart failure clinic visits during the three years prior to- and one year following the establishment of ATTR-CA were recorded as well.

### Statistical methods

The mean and SDs are presented, and a two-sided *t* test was performed to determine the statistical significance of differences in echocardiographic parameters for patients with and without ATTR-CA. For comparing categorical data between the two groups, the Chi-square test was used. Original data were also compared between the two groups using the nonparametric Mann–Whitney *U* test. P values <0.05 were considered significant. Statistical analysis was performed using IBM SPSS version 21.0 (Armonk, NY, USA).

## Results

**Fig 1:**
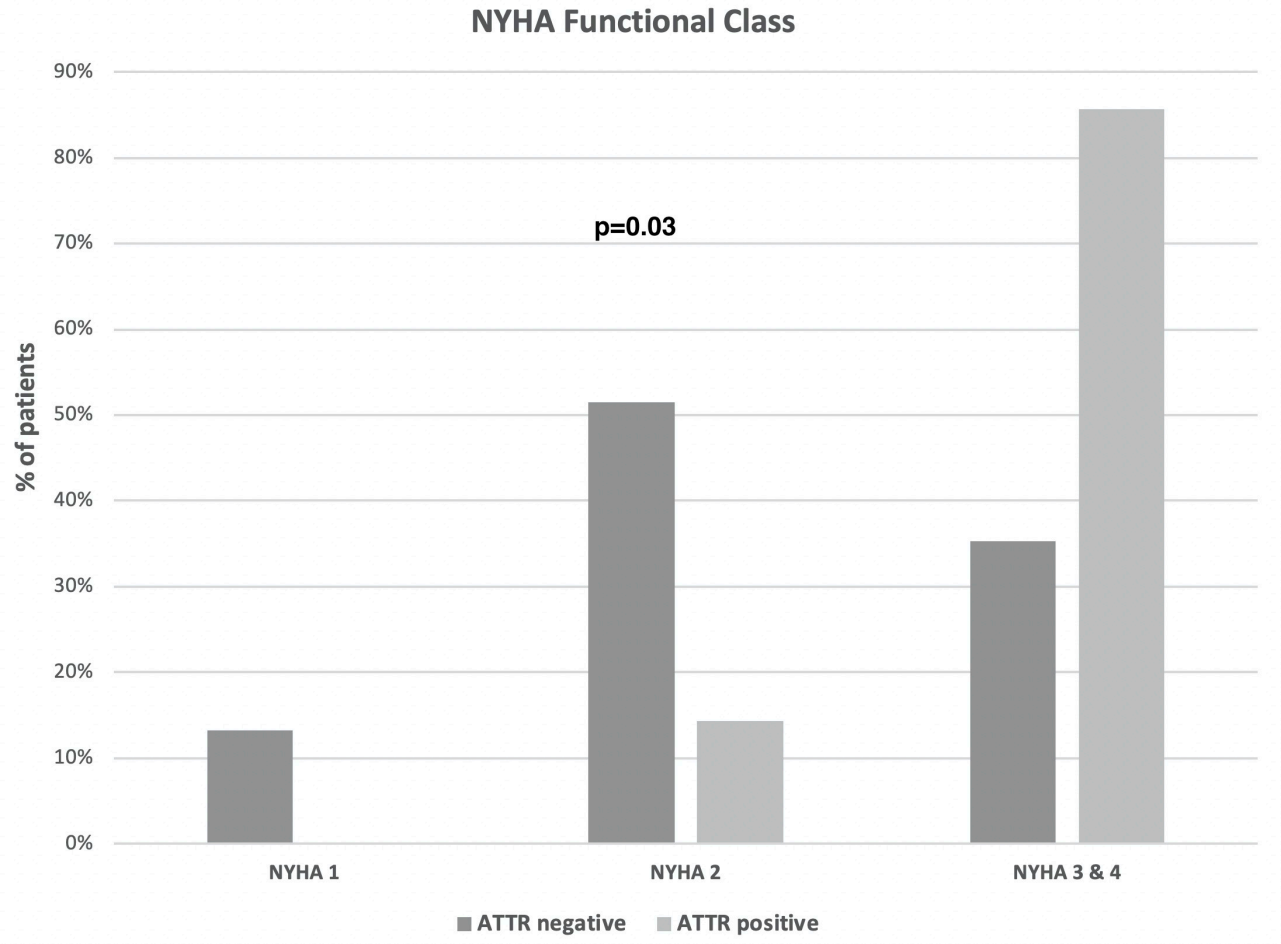
Comparison between patents with and without ATTR-CA according to the NYHA FC (NYHA FC, New York Heart Association Functional Class).

Out of 75 patients with heart failure of unexplained cause who underwent an extensive work up for ATTR, seven patients (9.3% (95% CI 5.6–12.4%) had both a positive planar ^99m^Tc-PYP and SPECT scintigraphy and AL amyloidosis was ruled out. Patient baseline characteristics and comparisons between patients with and without ATTR-CA are presented in Table 1. The two groups were similar in terms of comorbidities. However, patients with ATTR-CA were older (p = 0.01), there was a trend for higher prevalence of male gender (p = 0.07), and a significantly higher proportion of them had mild to moderate renal failure (GFR<60 ml/min) (57% vs. 12%, p = 0.01). All patients were treated following the recent guidelines for management of patients with systolic HF [17]. However, there was a trend for lower use of ACEI/ARB in patients with ATTR-CA (57% vs. 97%, p = 0.01), possibly due to impaired renal function or impaired tolerance due to hypotension. In addition, they had a greater need for oral furosemide treatment compared with patients without ATTR-CA. Regarding the functional status, the vast majority of patients with ATTR-CA were in NYHA class 3 and 4 compared with controls (85% vs. 35%, p = 0.03) representing a group with more advanced heart failure (Fig 1).

**Table 1:** Baseline characteristics of patients with and without ATTR-CA.

	ATTR-CA (n = 7)	No ATTR-CA(n = 68)	p-value
Age (years)	75.0±6.1	64.0±11.6	0.01
Sex, male (%)	100%	66%	0.07
Background			0.23
Ashkenazi Jews	5 (71%)	28 (41%)	
Middle East and North African background	2 (29%)	40 (59%)	
BMI (kg/m^2^)	30.1±6.4	29.1±6.4	0.60
Hypertension n (%)	7 (100)	48 (70.6)	0.12
DM n (%)	2 (29.5)	30 (44.8)	0.82
Obesity n (%)	2 (29.5)	30 (44.8)	0.59
Smoking n (%)	1 (14.3)	22 (32.3)	0.43
AF n (%)	4 (57.1)	20 (29.8)	0.14
CVA/TIA n (%)	0 (0.0)	4 (5.9)	0.83
CRF (GFR<60 mL/min) n (%)	4 (57.1)	8 (12.2)	0.01
CRT n (%)	1 (14.3)	18 (20.0)	0.80
Medical treatment			
ACEI/ARB/ARNI n (%)	4 (57.1)	66 (97.1)	0.10
Beta blockers n (%)	7 (100)	61 (89.7)	0.41
MRA n (%)	5 (71.4)	35 (52.2)	0.14
Diuretics n (%)	6 (85.7)	45 (66.2)	<0.01

BMI–basal metabolic index, DM–diabetes mellitus, AF–atrial fibrillation or atrial flutter, CVA–cerebrovascular accident, TIA–transient ischemic attack, CRT–cardiac resynchronization therapy, CRF–chronic renal failure, GFR–glomerular filtration rate, ACEI–angiotensin converting enzyme inhibitor, ARB–angiotensin receptor blocker, ARNI–angiotensin receptor and neprilysin inhibitor, MRA–mineralocorticoid receptor inhibitor.

### Echocardiography and electrocardiography

**Fig 2:**
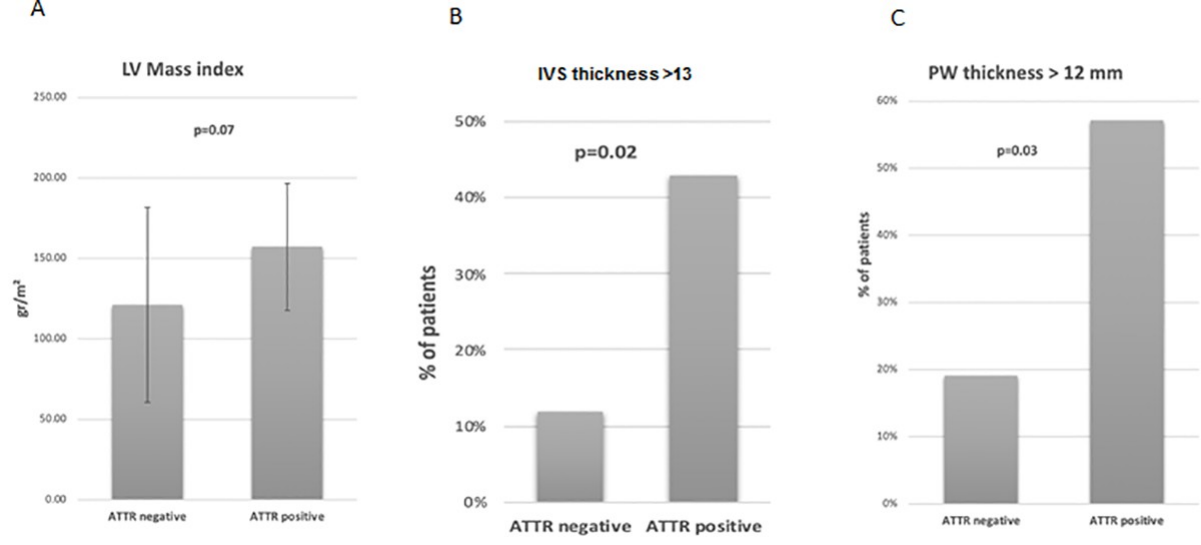
Comparison of patients’ left ventricular mass index (LVMi), interventricular septum (IVS) thickness, and posterior wall (PW) thickness. A. Left Ventricular Mass index (gr/m^2^). B. Interventricular Septum Thickness > 13 mm (%). C. Posterior Wall Thickness > 12 mm (%).

No significant differences were seen between patients with and without ATTR-CA with regard to LVEF, LV diastolic diameter and left atrial area (Table 2). Among ATTR-CA patients, increased wall thickness (interventricular septal {IVS} and posterior wall {PW}) (p<0.01 and p = 0.02, respectively) as well as increased LV mass index (p = 0.07) were evident. When looking at the prevalence of LVH, a significantly larger proportion of patients with ATTR-CA presented with LVH compared to those with no ATTR-CA (IVS >13 mm 57.1%. vs. 19.1% p = 0.02; PW > 12 mm 42.9% vs 11.8%, p = 0.03) (Fig 2). In addition, patients with positive scans had significantly higher E/e’ ratio (17.6±7.7 vs. 12.2±9.7, p = 0.06). In this study ECG features of LVH as defined by Sokolow index did not differ significantly between patients with positive and negative PYP scans (17±8 vs 17±9, p = 0.98) as well as Sokolow index/LV mass ratio (0.15±0.09 vs. 0.16 ±0.08, p = 0.68). However, ECG measures of LVH were obtained only in a small number of patients (in 4 patients with ATTR-CA and 40 patients without ATTR-CA) due to the high proportion of patients with pacemakers, CRT and RBBB, which made LVH measurement impossible.

**Table 2:** Comparison of echocardiographic measures between patents with and without ATTR-CA.

	ATTR-CA (n = 7)	No ATTR-CA (n = 68)	p-value
LV ejection fraction (%)	32.0 ± 8.3	36.2 ± 7.8	0.20
LV end diastolic diameter (mm)	50.9±7.0	53.0±8.0	0.50
IVS thickness >13 mm n (%)	4 (57.1)	13 (19.1)	0.02
IVS thickness (mm)	14±4.93	11.15±2.26	<0.01
PW thickness (mm)	12.14±3.53	9.90±2.16	0.02
LV Mass indexed (gr/m^2^)	157.1±60.6	121.0±39.5	0.07
LA area (cm^2^)	28.5 ± 9.2	24.2 ± 7.6	0.20
e’ (m/sec)	0.06±0.04	0.07±0.04	0.37
E/e’	17.6±7.7	12.2±9.7	0.06
Elevated Filling pressure n (%)	5 (71.4)	31 (45.6)	0.20
PAP (mmHg)	51.7 ± 11.5	39.7 ± 14.7	0.04
Global Longitudinal Strain average, %	-9.6 ± 2.1	-11.2 ± 3.8	0.17

LV–left ventricle, IVS–interventricular septum, PW–posterior wall, LA–left atrium, PAP–pulmonary artery pressure, AP–apical, MID–mid-wall, BAS–basal.

When analyzing 2DS parameters, a low LV longitudinal global strain (LG strain) was obtained in all patients, which is usually seen in patients with LV dysfunction. Patients with ATTR-CA had a trend towards a more impaired LG strain, but the difference did not reach statistical significance (−9.6±2.1 versus −11.2±3.8%, p = 0.17). An apical sparing index of >1 was obtained in only one patient with a positive scan and was not seen in patients with negative scans.

### Clinical course

**Fig 3:**
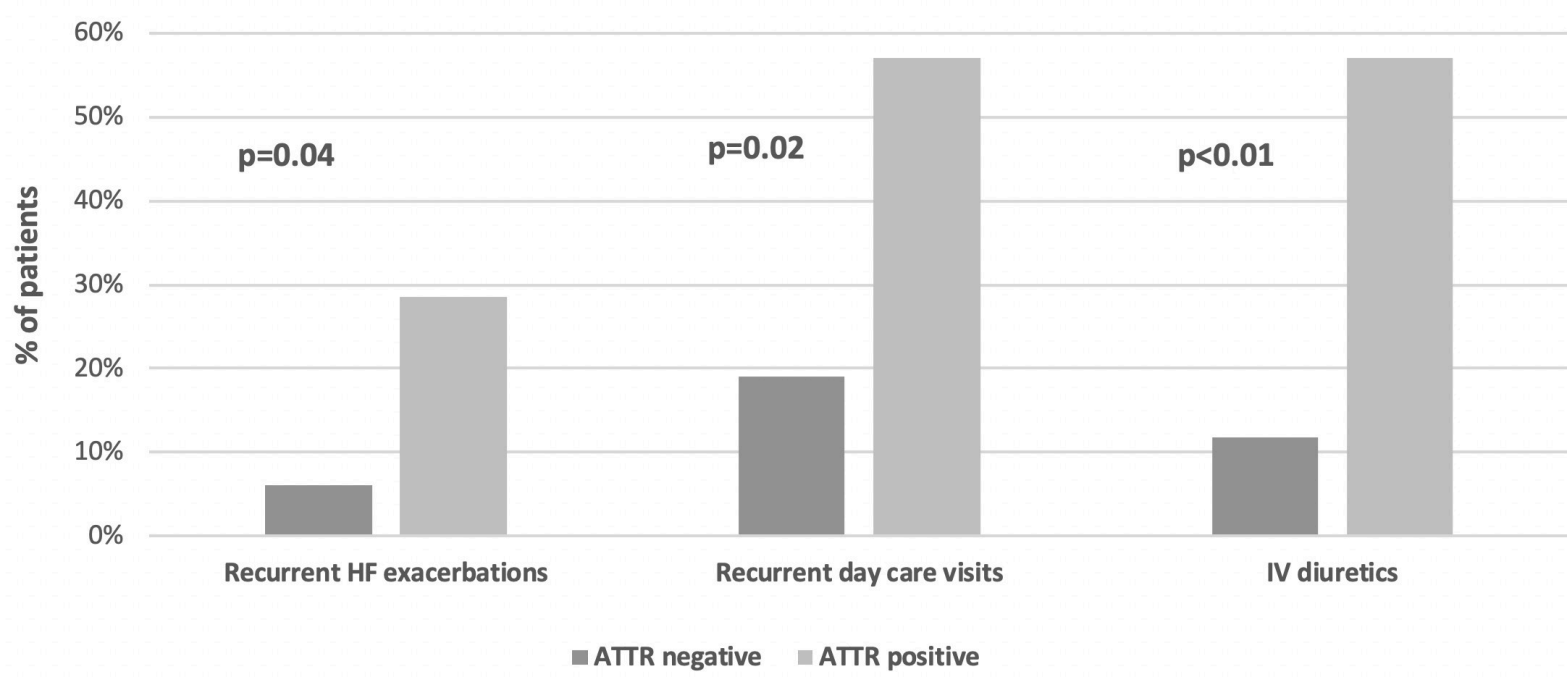
Comparison of clinical course between patents with and without ATTR-CA according to heart failure exacerbation events.

The analysis of HF worsening events during the last three years prior- and one-year post- recruitment showed that patients with ATTR-CA had a more severe clinical course of the disease (Fig 3) requiring admissions for recurrent HF exacerbations (29% vs. 6%, p = 0.04), and more visits to the heart failure day care unit for intravenous diuretic treatment (57% vs. 12%, p = 0.02) (Fig 3).

### Descriptive features of patients with positive ^99m^Tc-PYP scans

The main individual clinical and echocardiographic features of patients with ATTR-CA are presented in the Table 3. Three out of seven patients (patients 2, 3, and 6) had low voltage (<5 mm) on limb leads and one patient (patient 6) had apical sparing (index >1. Patients with relative apical sparing (index >0.8) had more prominent LVH (as defined by IVS thickness >13 mm and LV posterior wall thickness >12 mm). Patient 1 was treated by cardiac resynchronization therapy (CRT). Patients 2 and 3 had an implanted cardiac defibrillator (ICD). Patient 5 had a ventricular pacemaker without a defibrillator; he had atrial fibrillation with ventricular pacing. Patient 6 had the most typical signs of TTR amyloidosis and the most prominent apical sparing: a restrictive LV filling pattern, mean wall thickness of 18 mm, and mid-range LVEF. He also had a diagnosis of carpal tunnel syndrome, but not of true peripheral polyneuropathy. Genetic testing of TTR revealed no mutations in positive ^99m^Tc-PYP patients and AL amyloidosis was ruled out in all patients.

**Table 3:** 

	LVEF %	NYHA FC	HF exacerbation *	IVS (mm)	LVEDD (mm)	LV Mass indexed (gr/m^2^)	Grade DD	Apical sparing index **	Pacing	Rhythm	GFR (mL/min)	Peripheral neuropathy
**1**	37	3	Yes	14	57	157.8	1	0.45	CRT	CRT	49.8	No
**2**	30	3	Yes	6	45	51.7	2	0.34	none	sinus	47.9	No
**3**	25	2	No	13	51	160.5	1	0.47	none	sinus	36.1	No
**4**	25	3	Yes	10	63	143.9	3	0.42	none	sinus	81.2	No
**5**	25	3	Yes	20	46	198.5	3	0.82	RV	AF	43.7	No
**6**	45	3	No	19	50	230.4	3	1.34	none	AF	88.4	Yes
**7**	35	3	Yes	16	58	157.8	2	0.90	none	sinus	91.4	No

The individual clinical and echocardiographic features of patients with ATTR-CA.

*Need for recurrent hospitalization for heart failure, recurrent heart failure day care unit visit or IV furosemide.

** Apical longitudinal strain/(Mid-wall longitudinal strain + basal longitudinal strain).

LVEF–left ventricular ejection fraction, NYHA FC–New York Heart Association Function Class, IVS–interventricular septum, LVEDD–left ventricular end-diastolic diameter, LV–left ventricle, GFR–glomerular filtration rate by Cockroft Gault, CRT–cardiac resynchronization therapy, RV–right ventricle, AF–atrial fibrillation.

## Discussion

In our prospective study, we found for the first time that 9% of patients with HFrEF of unexplained etiology had a co-existing ATTR-CA confirmed by a ^99m^Tc-PYP SPECT imaging. had a co-existing ATTR-CA confirmed by a ^99m^Tc-PYP SPECT imaging. Notably, all but one had moderate to severe LV dysfunction. This is the first study to specifically and separately address the prevalence, echocardiographic and clinical features of ATTR-CA among this subpopulation of HF patients with unexplained LV dysfunction.

### Non-biopsy diagnosis of ATTR-CA in patients with reduced LV function

Gilmore et al. found very high sensitivity of a positive scan alone for detecting TTR amyloid (>99%), with a specificity of 86% in a large international study. Importantly, false-positive results were found mostly in AL amyloidosis patients [12]. In our study, AL amyloidosis was excluded in all patients. Diagnostic accuracy of bone scintigraphy in the assessment of ATTR-CM was studied in two recently published meta-analyses [23,24]. Sensitivities were 92.2% and 91.7%, while specificity was 95.4% and 95.8%, respectively. A very high positive likelihood ratio of 22 was calculated for Tc-PYP scintigraphy compared to any organ positive histology [24]. Assuming low 0.1 or 0.05 frequencies of true disease in our study population, we would still expect positive predictive value of 0.7 and 0.51, respectively. Currently, there is only one study of Tc-PYP scintigraphy using a CZT camera, with 100% sensitivity and specificity [25]. Despite the small sample size (n = 43), the calculated diagnostic accuracy was 100% (95% CI 91.8%-100%). In the current study, positive patients were verified by both conventional camera and CZT camera. In our study, we have supplemented all PYP scintigraphy studies with SPECT CT to rule out false positive. The use of SPECT CT is considered highly effective in eliminating the possibility of blood pool that would result in a false positive study [18,19]. We believe that using both the CZT camera and the addition of SPECT imaging is sufficient to establish the diagnosis of ATTR-CA in the absence of monoclonal gammopathy.

### Clinical characteristics and course of the disease

In line with previously published series of patients with wtATTR-CA, where >90% of patients were men in their 70’s [15,26,27], in our study, all patients with a non-biopsy diagnosis of wtATTR-CA were males and were significantly older compared with patients with no ATTR-CA. The clinical course of these ATTR CA patients is evident by more severe symptoms of HF and refractory course requiring IV diuretic treatment and a higher rate of admissions, despite a similar degree of LV dysfunction.

A number of studies described wtATTR-CA as a progressive disease with poor prognosis when untreated, reporting on ~ 3–4 years survival depending on the stage of the disease [10,15,16,28,29]. Ruberg et al. found median survival after diagnosis to be 43 months at a prospective follow up of 18 patients with wtATTR-CA and 11 with V122I hATTR [30]. Importantly, the mortality was preceded by a decrease in LVEF, an increase in biomarker levels, and worsening of their functional status, representing the progression of the disease. Similar results were reported in a recent prospective study that described post diagnosis survival of 46.7 months [26]. These findings support the perception that the clinical course of wtATTR–CA may remain relatively stable for years with subsequent rapid progression of heart failure leading to death [16]. Unfortunately, these patients are usually diagnosed when signs of refractory heart failure occur. An important step forward was done by introducing a staging of ATTR cardiomyopathy for risk stratification assessment. Increased cardiac biomarker levels such as troponin T and N-terminal pro–B-type natriuretic peptide and reduced GFR appear to be associated with reduced survival [12,15].

### The morphologic phenotype

ATTR cardiomyopathy is an under-recognized disease with increasing attention in the last decade. It is often misdiagnosed because of very similar phenotypes in hypertension, hypertrophic cardiomyopathy, and aortic stenosis, where all these conditions are accompanied by LV hypertrophy [13,31–33]. A list of clinical “red flags” was proposed to make a timely diagnosis [14,34] emphasizing that patients with LVH (≥14 mm) in the absence of hypertension accompanied by either HFpEF or other complimentary clinical clues should raise the clinical suspicion of ATTR-CA [28,35–37]. Gonzales-Lopez et al. reported wtATTR-CA in 13.3% of Caucasian patients over 60 years old with LV hypertrophy (wall thickness ≥12 mm) and HFpEF [34]. Similar data were published by Damy et al. who found a prevalence of ~11% of hATTR-CA among their male population older than 65 [38].

Although all patients in the current study had impaired systolic LV function, the structural primary phenotype of the patients with preexisting ATTR-CA was characterized by non-dilated left ventricles, LV hypertrophy, increased LV mass, and elevated pulmonary artery pressures on echocardiography in the majority of patients. Phenotypic presentations as either restrictive cardiomyopathy or HFpEF are considered the classical clinical variant of ATTR-CA. However, reduced LVEF has been described mostly in elderly patients with wtATTR-CA, and in those with the Val122Ile mutation [1,4,13,15,16,39]. Rapezzi et al. described three groups of patients with amyloidosis from two large Italian centers, 157 patients with AL cardiac amyloidosis (AL-CA), 61 with hATTR-CA and 15 with wtATTR-CA. All patients presented with non-dilated LV, and LVH which was most prominent in patients with wtATTR-CA. The LVEF differed between the groups, tending to be normal in hATTR-CA, lower normal limits in AL-CA, and abnormally low in wtATTR-CA. An LVEF <40% was found in 22% in AL-CA, 8% in hATTR-CA, and 40% in wtATTR-CA patients. Moreover, the majority of patients did not display a restrictive filling pattern considered as a key finding in this patient population. Grogan et al. published a retrospective review of 360 patients from the Mayo Clinic with a diagnosis of wtATTR-CA [15]. These patients had a markedly elevated LV mass index (median: 160 g/m^2^; IQR: 132 to 195 g/m^2^) with restrictive filling (median E/A: 2.0, IQR: 1.0 to 3.0). Notably, an LVEF at diagnosis <50% was present in 151 patients (45%) and <40% in 93 (27.9%). In line with this report, Golzalez-Lopez et al. showed that out of 108 patients with wtATTR-CA, 37% had an LVEF < 50% at diagnosis, and 9% of the patients had an LVEF of < 30% [13]. Recently, Lopez-Sainz et al. described a small group of patients admitted with unexplained systolic HF (LVEF<50%) and LVH [39]. Out of 28 patients (mean age 78 ± 9, mean LVEF was 41 ± 7%, 17 with HFmrEF and 9 with HFrEF) screened for ATTR using ^99m^Tc-DPD scintigraphy three (11%) were positive for wtATTR. In the present study we found a meaningful prevalence (9%) of wtATTR patients with HF and an unclear cause of non-ischemic cardiomyopathy with impaired LV function. Of note, in our study, all but one patient presented with moderate to severe LV dysfunction (LVEF ≤35%) and more than half had an LVEF ≤30% (25–30%) representing a group of patients with HFrEF, but not HFmrEF as described in most of the studies. Importantly, despite similar LVEF in both groups, patients with ATTR-CA had increased mean wall thickness, LV mass index, and higher pulmonary pressures. However, when looking at the individual data, although LVH appeared to be particularly frequent, two patients did not have LVH and an additional two patients had some degree of LV dilation and did not have elevated LV filling pressure upon echocardiography. In addition, a low-voltage electrocardiographic pattern was present only in approximately half of the patients. Moreover, although the average LGS tended to be lower in patients with wtATTR-CA, an apical spearing index >1 was present only in one patient with an LVEF of 45% and absent in those with LVEF<40%. The lack of a consistent presence of an apical sparing, which was described mostly in patients with HFpEF or HFmrEF [22], may be related to severely depressed LV function in most of the patients. Based on our observation, in individual patients with significantly depressed LV function, these typical measures cannot be used as an indication for ATTR-CA screening.

The question as to whether the presence of ATTR is pathogenically related to the compromised LV function and HF cannot be answered by this study. Traditionally, extracellular deposition of misfolded aggregated TTR is viewed as a space occupying process that impairs cardiac relaxation. However, we have recently shown, that aggregated TTR has a direct proapoptotic/toxic effect on cardiomyocytes suggesting that long term in-situ exposure could reduce cardiac mass and result in reduced function [40].

In conclusion, our findings that a meaningful percentage of patients with unexplained LV dysfunction have a co-existing ATTR-CA suggests that we should consider widening the margins of clinical suspicion for this disease, especially in light of the currently approved TTR stabilizer and the wealth of silencers that are in advanced clinical testing.

### Clinical implications

Other than the expansion of the clinical spectrum related to ATTR-CA, there are other interesting potential implications to the current study. ATTR-CA with reduced systolic function could represent the natural progression from an intrinsic and restrictive type, to a ’burnt out’ phase of the cardiomyopathy. If this is indeed the case, the latter presentation is expected to be more malignant and thus initial identification and treatment may be of special importance as a dilated pattern is less likely to be influenced by disease modifying agents.

## Limitations

The main limitation of this prospective study is a relatively small number of patients.

However, all patients enrolled in this prospective study underwent an extensive workup including a detailed history and physical examination, electrocardiography, and echocardiography. Those patients with a positive 99m Tc-PYP scintigraphy underwent genetic screening and additional workup to exclude primary amyloidosis (AL) (serum free light chain essay and serum/urine immunofixation). The number of patients with a positive scan is low to allow a robust comparison to the patients with a negative scan. However, it should be noted that this study is a primary descriptive study to raise awareness of the heterogeneity of ATTR-CA followed by the diagnostic workup of patients with systolic heart failure and subsequent clinical management.

## References

[1] Rapezzi C, Merlini G, Quarta CC, Riva L, Longhi S, Leone O, et al. Systemic cardiac amyloidoses: disease profiles and clinical courses of the 3 main types. Circulation. 2009;120(13):1203–12. doi: 10.1161/CIRCULATIONAHA.108.843334 19752327

[2] Ruberg FL, Berk JL. Transthyretin (TTR) cardiac amyloidosis. Circulation. 2012;126(10):1286–300. doi: 10.1161/CIRCULATIONAHA.111.078915 22949539 PMC3501197

[3] Maurer MS, Elliott P, Comenzo R, Semigran M, Rapezzi C. Addressing Common Questions Encountered in the Diagnosis and Management of Cardiac Amyloidosis. Circulation. 2017;135(14):1357–77. doi: 10.1161/CIRCULATIONAHA.116.024438 28373528 PMC5392416

[4] Gonzalez-Lopez E, Lopez-Sainz A, Garcia-Pavia P. Diagnosis and Treatment of Transthyretin Cardiac Amyloidosis. Progress and Hope. Rev Esp Cardiol (Engl Ed). 2017;70(11):991–1004. doi: 10.1016/j.rec.2017.05.036 28870641

[5] Rapezzi C, Lorenzini M, Longhi S, Milandri A, Gagliardi C, Bartolomei I, et al. Cardiac amyloidosis: the great pretender. Heart Fail Rev. 2015;20(2):117–24. doi: 10.1007/s10741-015-9480-0 25758359

[6] Gillmore JD, Damy T, Fontana M, Hutchinson M, Lachmann HJ, Martinez-Naharro A, et al. A new staging system for cardiac transthyretin amyloidosis. Eur Heart J. 2018;39(30):2799–806. doi: 10.1093/eurheartj/ehx589 29048471

[7] Manolis AS, Manolis AA, Manolis TA, Melita H. Cardiac amyloidosis: An underdiagnosed/underappreciated disease. Eur J Intern Med. 2019;67:1–13. doi: 10.1016/j.ejim.2019.07.022 31375251

[8] Siddiqi OK, Ruberg FL. Cardiac amyloidosis: An update on pathophysiology, diagnosis, and treatment. Trends Cardiovasc Med. 2018;28(1):10–21. doi: 10.1016/j.tcm.2017.07.004 28739313 PMC5741539

[9] Siepen FAD, Bauer R, Voss A, Hein S, Aurich M, Riffel J, et al. Predictors of survival stratification in patients with wild-type cardiac amyloidosis. Clin Res Cardiol. 2018;107(2):158–69. doi: 10.1007/s00392-017-1167-1 28956153

[10] Lane T, Fontana M, Martinez-Naharro A, Quarta CC, Whelan CJ, Petrie A, et al. Natural History, Quality of Life, and Outcome in Cardiac Transthyretin Amyloidosis. Circulation. 2019;140(1):16–26. doi: 10.1161/CIRCULATIONAHA.118.038169 31109193

[11] Patel KS, Hawkins PN. Cardiac amyloidosis: where are we today? J Intern Med. 2015;278(2):126–44. doi: 10.1111/joim.12383 26077367

[12] Gillmore JD, Maurer MS, Falk RH, Merlini G, Damy T, Dispenzieri A, et al. Nonbiopsy Diagnosis of Cardiac Transthyretin Amyloidosis. Circulation. 2016;133(24):2404–12. doi: 10.1161/CIRCULATIONAHA.116.021612 27143678

[13] Gonzalez-Lopez E, Gagliardi C, Dominguez F, Quarta CC, de Haro-Del Moral FJ, Milandri A, et al. Clinical characteristics of wild-type transthyretin cardiac amyloidosis: disproving myths. Eur Heart J. 2017;38(24):1895–904. doi: 10.1093/eurheartj/ehx043 28329248

[14] Maurer MS, Bokhari S, Damy T, Dorbala S, Drachman BM, Fontana M, et al. Expert Consensus Recommendations for the Suspicion and Diagnosis of Transthyretin Cardiac Amyloidosis. Circ Heart Fail. 2019;12(9):e006075. doi: 10.1161/CIRCHEARTFAILURE.119.006075 31480867 PMC6736650

[15] Grogan M, Scott CG, Kyle RA, Zeldenrust SR, Gertz MA, Lin G, et al. Natural History of Wild-Type Transthyretin Cardiac Amyloidosis and Risk Stratification Using a Novel Staging System. J Am Coll Cardiol. 2016;68(10):1014–20. doi: 10.1016/j.jacc.2016.06.033 27585505

[16] Ruberg FL, Grogan M, Hanna M, Kelly JW, Maurer MS. Transthyretin Amyloid Cardiomyopathy: JACC State-of-the-Art Review. J Am Coll Cardiol. 2019;73(22):2872–91. doi: 10.1016/j.jacc.2019.04.003 31171094 PMC6724183

[17] Ponikowski P, Voors AA, Anker SD, Bueno H, Cleland JGF, Coats AJS, et al. 2016 ESC Guidelines for the diagnosis and treatment of acute and chronic heart failure: The Task Force for the diagnosis and treatment of acute and chronic heart failure of the European Society of Cardiology (ESC)Developed with the special contribution of the Heart Failure Association (HFA) of the ESC. Eur Heart J. 2016;37(27):2129–200. doi: 10.1093/eurheartj/ehw128 27206819

[18] Dorbala S, Ando Y, Bokhari S, Dispenzieri A, Falk RH, Ferrari VA, et al. ASNC/AHA/ASE/EANM/HFSA/ISA/SCMR/SNMMI expert consensus recommendations for multimodality imaging in cardiac amyloidosis: Part 2 of 2-Diagnostic criteria and appropriate utilization. J Nucl Cardiol. 2019. doi: 10.1016/j.cardfail.2019.08.002 31468377

[19] Dorbala S, Ando Y, Bokhari S, Dispenzieri A, Falk RH, Ferrari VA, et al. ASNC/AHA/ASE/EANM/HFSA/ISA/SCMR/SNMMI Expert Consensus Recommendations for Multimodality Imaging in Cardiac Amyloidosis: Part 1 of 2-Evidence Base and Standardized Methods of Imaging. J Card Fail. 2019;25(11):e1–e39. doi: 10.1016/j.cardfail.2019.08.001 31473268

[20] Devereux RB, Alonso DR, Lutas EM, Gottlieb GJ, Campo E, Sachs I, et al. Echocardiographic assessment of left ventricular hypertrophy: comparison to necropsy findings. Am J Cardiol. 1986;57(6):450–8. doi: 10.1016/0002-9149(86)90771-x 2936235

[21] Lang RM, Badano LP, Mor-Avi V, Afilalo J, Armstrong A, Ernande L, et al. Recommendations for cardiac chamber quantification by echocardiography in adults: an update from the American Society of Echocardiography and the European Association of Cardiovascular Imaging. Eur Heart J Cardiovasc Imaging. 2015;16(3):233–70. doi: 10.1093/ehjci/jev014 25712077

[22] Phelan D, Collier P, Thavendiranathan P, Popovic ZB, Hanna M, Plana JC, et al. Relative apical sparing of longitudinal strain using two-dimensional speckle-tracking echocardiography is both sensitive and specific for the diagnosis of cardiac amyloidosis. Heart. 2012;98(19):1442–8. doi: 10.1136/heartjnl-2012-302353 22865865

[23] Treglia G, Glaudemans A, Bertagna F, Hazenberg BPC, Erba PA, Giubbini R, et al. Diagnostic accuracy of bone scintigraphy in the assessment of cardiac transthyretin-related amyloidosis: a bivariate meta-analysis. Eur J Nucl Med Mol Imaging. 2018;45(11):1945–55. doi: 10.1007/s00259-018-4013-4 29687207

[24] Brownrigg J, Lorenzini M, Lumley M, Elliott P. Diagnostic performance of imaging investigations in detecting and differentiating cardiac amyloidosis: a systematic review and meta-analysis. ESC Heart Fail. 2019;6(5):1041–51. doi: 10.1002/ehf2.12511 31487121 PMC6816075

[25] Flaherty KR, Morgenstern R, Pozniakoff T, DeLuca A, Castano A, Maurer MS, et al. (99m)Technetium pyrophosphate scintigraphy with cadmium zinc telluride cameras is a highly sensitive and specific imaging modality to diagnose transthyretin cardiac amyloidosis. J Nucl Cardiol. 2020;27(2):371–80. doi: 10.1007/s12350-019-01831-8 31463816

[26] Connors LH, Sam F, Skinner M, Salinaro F, Sun F, Ruberg FL, et al. Heart Failure Resulting From Age-Related Cardiac Amyloid Disease Associated With Wild-Type Transthyretin: A Prospective, Observational Cohort Study. Circulation. 2016;133(3):282–90. doi: 10.1161/CIRCULATIONAHA.115.018852 26660282 PMC4718760

[27] Pinney JH, Whelan CJ, Petrie A, Dungu J, Banypersad SM, Sattianayagam P, et al. Senile systemic amyloidosis: clinical features at presentation and outcome. J Am Heart Assoc. 2013;2(2):e000098. doi: 10.1161/JAHA.113.000098 23608605 PMC3647259

[28] Damy T, Kristen AV, Suhr OB, Maurer MS, Plante-Bordeneuve V, Yu CR, et al. Transthyretin cardiac amyloidosis in continental Western Europe: an insight through the Transthyretin Amyloidosis Outcomes Survey (THAOS). Eur Heart J. 2019. doi: 10.1093/eurheartj/ehz173 30938420 PMC8825236

[29] Narotsky DL, Castano A, Weinsaft JW, Bokhari S, Maurer MS. Wild-Type Transthyretin Cardiac Amyloidosis: Novel Insights From Advanced Imaging. Can J Cardiol. 2016;32(9):1166.e1-.e10. doi: 10.1016/j.cjca.2016.05.008 27568874 PMC5004088

[30] Ruberg FL, Maurer MS, Judge DP, Zeldenrust S, Skinner M, Kim AY, et al. Prospective evaluation of the morbidity and mortality of wild-type and V122I mutant transthyretin amyloid cardiomyopathy: the Transthyretin Amyloidosis Cardiac Study (TRACS). Am Heart J. 2012;164(2):222–8.e1. doi: 10.1016/j.ahj.2012.04.015 22877808

[31] Castano A, Narotsky DL, Hamid N, Khalique OK, Morgenstern R, DeLuca A, et al. Unveiling transthyretin cardiac amyloidosis and its predictors among elderly patients with severe aortic stenosis undergoing transcatheter aortic valve replacement. Eur Heart J. 2017;38(38):2879–87. doi: 10.1093/eurheartj/ehx350 29019612 PMC5837725

[32] Galat A, Guellich A, Bodez D, Lipskaia L, Moutereau S, Bergoend E, et al. Causes and consequences of cardiac fibrosis in patients referred for surgical aortic valve replacement. ESC Heart Fail. 2019;6(4):649–57. doi: 10.1002/ehf2.12451 31115164 PMC6676299

[33] Ternacle J, Krapf L, Mohty D, Magne J, Nguyen A, Galat A, et al. Aortic Stenosis and Cardiac Amyloidosis: JACC Review Topic of the Week. J Am Coll Cardiol. 2019;74(21):2638–51. doi: 10.1016/j.jacc.2019.09.056 31753206

[34] Gonzalez-Lopez E, Gallego-Delgado M, Guzzo-Merello G, de Haro-Del Moral FJ, Cobo-Marcos M, Robles C, et al. Wild-type transthyretin amyloidosis as a cause of heart failure with preserved ejection fraction. Eur Heart J. 2015;36(38):2585–94. doi: 10.1093/eurheartj/ehv338 26224076

[35] Yamamoto H, Yokochi T. Transthyretin cardiac amyloidosis: an update on diagnosis and treatment. ESC Heart Fail. 2019. doi: 10.1002/ehf2.12518 31553132 PMC6989279

[36] Witteles RM, Bokhari S, Damy T, Elliott PM, Falk RH, Fine NM, et al. Screening for Transthyretin Amyloid Cardiomyopathy in Everyday Practice. JACC Heart Fail. 2019;7(8):709–16. doi: 10.1016/j.jchf.2019.04.010 31302046

[37] Kittleson MM, Maurer MS, Ambardekar AV, Bullock-Palmer RP, Chang PP, Eisen HJ, et al. Cardiac Amyloidosis: Evolving Diagnosis and Management: A Scientific Statement From the American Heart Association. Circulation. 2020;142(1):e7–e22. doi: 10.1161/CIR.0000000000000792 32476490

[38] Damy T, Costes B, Hagege AA, Donal E, Eicher JC, Slama M, et al. Prevalence and clinical phenotype of hereditary transthyretin amyloid cardiomyopathy in patients with increased left ventricular wall thickness. Eur Heart J. 2016;37(23):1826–34. doi: 10.1093/eurheartj/ehv583 26537620

[39] Lopez-Sainz A, de Haro-Del Moral FJ, Dominguez F, Restrepo-Cordoba A, Amor-Salamanca A, Hernandez-Hernandez A, et al. Prevalence of cardiac amyloidosis among elderly patients with systolic heart failure or conduction disorders. Amyloid. 2019;26(3):156–63. doi: 10.1080/13506129.2019.1625322 31210553

[40] George J, Rappaport M, Shimoni S, Goland S, Voldarsky I, Fabricant Y, et al. A novel monoclonal antibody targeting aggregated transthyretin facilitates its removal and functional recovery in an experimental model. Eur Heart J. 2019. 10.1093/eurheartj/ehz695 31865366

